# Tetra-Primer Amplification-Refractory Mutation System (ARMS)—PCR for Genotyping Mouse Leptin Gene Mutation

**DOI:** 10.3390/ani12192680

**Published:** 2022-10-05

**Authors:** Jiangang Chen, Xinyun Xu, Paul Dalhaimer, Ling Zhao

**Affiliations:** 1 Department of Public Health, The University of Tennessee, Knoxville, TN 37996, USA; 2 Department of Nutrition, The University of Tennessee, Knoxville, TN 37996, USA; 3 Department of Chemical and Biomolecular Engineering, The University of Tennessee, Knoxville, TN 37996, USA

**Keywords:** leptin gene, tetra-primer amplification-refractory mutation system, heterozygous, genotyping, *ob*/*ob* mouse

## Abstract

**Simple Summary:**

We developed a method to detect a single-point mutation in an obese gene in a mouse research model. The method is quick, easy to conduct, and suitable for obesity and diabetes-related research, especially in settings with constrained resources to identify and differentiate wild-type mice from mice carrying a gene mutation.

**Abstract:**

Due to spontaneous deficiency in leptin, *ob*/*ob* mice are one of the most commonly used experimental animal models in diabetes research. In this study, we reported a quick and easy-to-conduct genotyping method using tetra-primer amplification refractory mutation system-polymerase chain reaction (ARMS-PCR) to differentiate mice with a mutated allele from the wild-type genotype. The amplicon patterns of different genotypes are clearly visible and distinguishable on 1.5% agarose gel. This method can serve as a valuable tool to differentiate genotypes for breeding purposes, to maintain animal colonies, control the available space in the animal facility, and identify appropriate individuals for animal experiments.

## 1. Introduction

Animal models are indispensable tools for researchers to understand the etiology, pathogenesis, progression, and to test novel strategies for treating and preventing metabolic disorders, including diabetes. *Ob*/*ob* mice, due to spontaneous deficiency in the leptin, are one of the most commonly used experimental animal models in diabetes research [1]. Leptin is a circulating peptide produced primarily by white adipocytes [2]. Encoded by the leptin (aka obese) gene, leptin regulates food intake, appetitive behaviors, energy homeostasis, skeletal growth, and reproduction [3,4]. The full-length leptin has a molecular weight of 16 kDa and is a 167 amino acid peptide with a 21 amino acid secretory signal sequence [5]. Upon binding to its cognate receptor, leptin initiates complex intracellular signaling cascades dictating various physiological processes, including metabolic and reproductive functions [6]. Leptin levels in circulation are in proportion to the amount of body fat, indicating the status of long-term energy stores [7].

A spontaneous single recessive mutation of the C to T substitution in codon 105 of the leptin gene leads to a premature stop codon and, subsequently, the absence of functional leptin peptide [8]. Mice that are homozygous for the Lep*^ob^* mutation (*ob*/*ob* mice) are obese with hyperphagia, transient hyperglycemia, glucose intolerance, and insulin resistance [9]. Although *ob*/*ob* males can occasionally reproduce if maintained on a restricted diet, *ob*/*ob* females are always infertile [10]. Therefore, genotyping is an essential tool to differentiate offspring genotypes for breeding purposes to maintain the animal colony, control the available space in the animal facility, and identify appropriate individuals for animal experiments [11,12].

In this study, we reported a tetra-primer ARMS-PCR method to detect a single leptin mutation using genomic DNA collected from mice’s tails. Tetra-primer ARMS-PCR has been applied to identify the single nucleotide polymorphism (SNP) [13,14,15] and differentiate whether the single mutation in DNA is heterozygous or homozygous [12,16,17]. This method takes advantage of the relative inability of Taq DNA polymerase to extend primers mismatched at their 3′-end and its intrinsic propensity of lacking 3′ to 5′ exonuclease activity, so the extension is dramatically reduced [18,19]. In the tetra-primer ARMS-PCR assay, the outer primers are non-allele-specific and used to amplify the region that comprises the SNP [20]. For inner primers, the last nucleotide at the 3′ end of the primer is designed to be complementary to the target nucleotide. An additional mismatch within the three bases closest to the SNP site at the 3′ end of the inner primer is purposely introduced [21,22]. This extra mismatch destabilizes the base pairing between the primers and their corresponding non-target templates, significantly improving the assay’s specificity by eliminating false-positive results [12,19,20,21]. The method is simple, robust, reliable, and requires no DNA purification. The method can distinguish heterozygotes from homozygotes for either allele in as little as 12.5 µL of PCR reaction in less than 2 h.

## 2. Materials and Methods

### 2.1. Animals

Mice that are heterozygous for the *Lep^ob^* mutation (B6.Cg-Lep*^ob^*/J, Strain #: 000632) and heterozygous for the leptin receptor diabetes spontaneous mutation (Lepr*^db^*) (B6.BKS(D)-Lepr*^db^*/J, Strain #: 000697) were purchased from the Jackson Laboratory (Bar Harbor, ME, USA). All mice were maintained in Mossman animal facility at the University of Tennessee, Knoxville. The animal protocol was approved by the University of Tennessee IACUC committee (approved #: 2320, 13 June 2022).

### 2.2. DNA Extraction from Tail Tissue

Crude DNA extraction from the tails of mice was conducted as described by Peng et al. [12]. Briefly, for each animal, approximately 1 mm of mouse tail tissue was removed and placed into 1.5 mL DNase, RNase-free tubes containing 180 μL of lysis buffer (50 mmol/L NaOH). The tubes were heated to 95 °C for 10 min, then put on ice for 1 min. After that, 20 μL of 1 mol/L Tris-HCl (pH 8.0) was added to each tube to neutralize the lysis buffer. The tubes containing samples were inverted 6 times, followed by centrifugation at 10,000 rpm at room temperature for 3 min to pellet the tissue debris, and the supernatant from each tube was used for the tetra-primer ARMS-PCR. The concentrations of DNA in the crude extraction supernatant were in the range of 14 to 18 ng/µL.

### 2.3. Primer Design and ARMS-PCR Parameters

The primers were designed based on the published leptin cDNA sequence of *mus musculus* strain C57BL/6J (GenBank: HQ166716.1). The sequences of the four primers used in this study are listed in Table 1.

**Figure 1:**
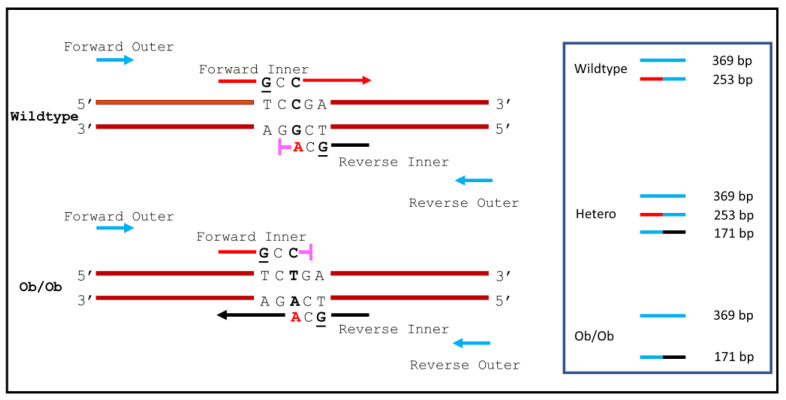
Diagram of the DNA amplicon patterns representing different genotypes on the agarose gel. Different colors indicate different primers participating in the PCR reaction. The two outer primers (blue-colored) are annealed at locations with different distances from the SNP site to ensure the production of two allele-specific PCR amplicons of different sizes distinguishable on the agarose gel. The base “G” (underlined) in the Forward Inner primer (red) and the Reverse Inner primer (black) were purposely introduced at position −2 from the 3′ terminal of each allele-specific primer to increase the specificity of the PCR reaction. 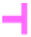: Refractory to the mutation, therefore the PCR reaction cannot proceed.

A schematic presentation of tetra-primer ARMS-PCR and the positions of PCR amplicons are described in Figure 1.

Tetra-primer ARMS-PCR was carried out with the Bio-Rad C1000 Touch^TM^ Thermal Cycler (Model No. CFX96^TM^ Optics Module, Bio-Rad, Hercules, CA, USA). Briefly, 12.5 µL of PCR reaction contained 2 μL of crude extraction supernatant (approximately 28–36 ng of DNA), 6.25 μL of DreamTaq 2× PCR master mix (ThermoFisher Scientific, Waltham, MA, USA), 0.25 µL of 10 μM of primer each (final concentration of 0.2 μmol/L). The PCR parameters were: Initial denaturation at 95 °C for 3 min, followed by 33 cycles of denaturation at 95 °C for 30 s, annealing at 58 °C for 30 s, and extension at 72 °C for 1 min. The final elongation step was 72 °C for 7 min. The amplicons were electrophoresed on a 1.5% agarose gel with ethidium bromide staining. The image was captured by Bio-Rad ChemiDoc XRS+ with Image Lab image acquisition and analysis software (Image Lab^TM^ Software, Version 5.2.1, Bio-Rad, Hercules, CA, USA).

### 2.4. Sequencing of Tetra-Primer ARMS-PCR Amplicons

Two separate PCR reactions were conducted (FI and RO pair to amplify the C allele, FO and RI pair to amplify the T allele). PCR bands corresponding to 253 bp and 171 bp were cut out from the gel, purified with the Zymoclean Gel DNA Recovery Kit (Zymo Research, Irvine, CA, USA), and sequenced by the Genomics Core Facility at the University of Tennessee, Knoxville, using an Applied Biosystem 3730 DNA Analyzer (Waltham, MA, USA) following Applied Biosystems BigDye Terminator v3.1 (Waltham, MA, USA) recommendations.

## 3. Results and Discussion

We have designed two sets of primers to amplify allele-specific PCR products from wildtype, heterozygous, and homozygous *ob*/*ob* mice using ARMS-PCR assays. A 253 bp PCR product is expected to be amplified from the wildtype allele by the Forward Inner and Reverse Outer primers (Figure 1). In contrast, a 171 bp PCR amplicon is expected to be amplified from the homozygous *ob*/*ob* allele by the Forward Outer and Reverse Inner primers. In addition, a 369 bp PCR product is also expected from both alleles by the Forward Outer and Reverse Outer primers as an internal control. Three PCR products differ significantly in length, making them easier to be differentiated by agarose gel electrophoresis.

Many factors need to be considered in developing a successful tetra-primer AMRS-PCR for SNP detection, including DNA extraction methods, annealing temperature (TA), PCR cycles, reagents, and primer concentrations. Among those, the TA is considered to be the most important [20].

**Figure 2:**
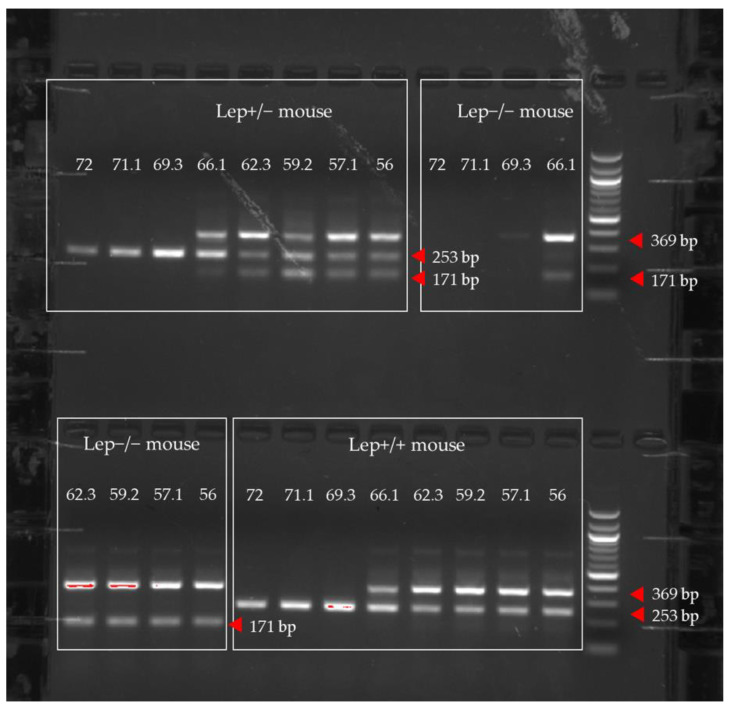
DNA samples collected from wildtype (Lep+/+), heterozygous (Lep+/−), and *ob*/*ob* mice (Lep−/−) were analyzed in a gradient tetra-primer ARMS-PCR (from 56 °C to 72 °C) to identify an optimal TA for the assay. Quick-Load^®^ 100 bp DNA Ladder (New England Biolabs, Ipswich, MA, USA) was used as the reference to facilitate the visualization of the positions of the amplicons on the 1.5% agarose gel. Red arrows correspond to the different sizes of PCR amplicons on the gel.

We conducted a gradient PCR to determine the impact of TA on the amplicon gel patterns (Figure 2).

As shown in Figure 2, when all other factors are held constant at a TA higher than 66.1 °C, no outer band (369 bp) was detected on the gel. The outer band in a tetra-primer ARMS-PCR serves as an internal control to indicate the quality of DNA samples and the performance of the tetra-primer ARMS-PCR reaction [16]. When TA was set between 56 and 66.1 °C, not only the outer band with the expected size was detected, but also clear allelic discrimination was visualized. Based on these data, we chose to set the TA to be 58 °C for the subsequent experiments.

**Figure 3:**
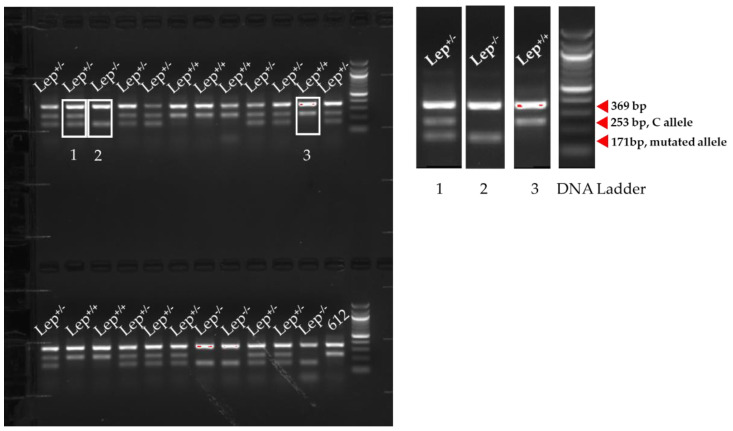
Tetra-primer ARMS-PCR was conducted on DNA samples collected from mice, followed by gel electrophoresis. Each lane represented one specific sample. Quick-Load^®^ 100 bp DNA Ladder (New England Biolabs, Ipswich, MA, USA) was used as the reference to visualize the difference in the positions of the amplicons on a 1.5% agarose gel. A zoomed insert was provided to elucidate the size and pattern of PCR amplicons corresponding to three different genotypes. 1.Lep+/−: heterozygous mouse carrying a mutated allele (T allele, 171 bp), 2. Lep−/−: *ob*/*ob* mouse (carrying T on both alleles), 3. Lep+/+: wildtype mouse (carrying C on both alleles, 253 bp). Red arrow indicates the size of specific band on the gel. Sample 612 was collected from a db/db mouse, which served as a wild-type control for the leptin gene.

We used the established tetra-primer ARMS-PCR and genotyped a total of 24 mice produced by heterozygous mouse breeding. As demonstrated in Figure 3, our tetra-primer ARMS-PCR generated three different amplicon patterns on the agarose gel that differentiated wildtype (Lep+/+), heterozygous (Lep+/−), and *ob*/*ob* (Lep−/−) genotypes. The sizes of each amplicon on the gel were easily identified and in agreement with the projection shown in Figure 1. DNA from mouse #612 was collected from a *db*/*db* mouse. Similar to *ob*/*ob* mice [23], *db*/*db* mice develop obesity and diabetes at a young age [24]. This strain of *db*/*db* mice has been backcrossed to the C57BL/6J genetic background [23,25], but unlike *ob*/*ob* mice, *db*/*db* mice have a leptin receptor gene mutation instead of a leptin gene mutation. Therefore, mouse #612 served as a “leptin wildtype” control in our study.

**Figure 4:**
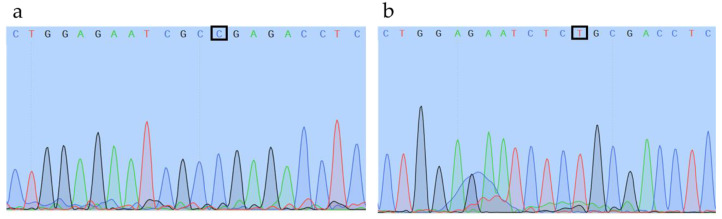
Sequencing results of the 253 bp and 171 bp amplicons. Gel bands corresponding to the size of 253 bp and 171 bp from three Lep+/− mice were cut out and pooled based on their size, respectively. PCR amplicons were then purified from the gel with Zymoclean Gel DNA Recovery Kit (Zymo Research, Irvine, CA, USA), and sequenced using Applied Biosystem 3730 DNA Analyzer (Waltham, MA, USA) following an Applied Biosystems BigDye Terminator v3.1 recommendations. (**a**) sequencing result of amplicon band size of 253 bp using Reverse Outer as the primer. Cytosine in the box indicates the C allele (wildtype); (**b**) sequencing result of an amplicon band size of 171 bp using Forward Outer as the primer. Thymine in the box indicates the substitution of C to T (mutant).

The genotype represented by the amplicon gel pattern of T/T (i.e., homozygous for the *ob*/*ob* allele) was further verified by the mice’s phenotype as they were substantially heavier than their counterparts. Moreover, to verify if the inner products of the 253 bp amplicon and 171 bp amplicon represent wildtype and *ob*/*ob* alleles, respectively, we cut out the corresponding bands from the gel and sequenced the amplicons. Figure 4a shows the sequencing result of the 253 bp amplicon (C allele, wildtype); Figure 4b shows the sequencing result of the 171 bp amplicon, which carried a C to T point mutation (mutant allele). Therefore, allele sequencing results verified the correct genotypes obtained by the tetra-primer AMRS-PCR method.

To further validate the tetra-primer ARMS-PCR genotyping method, DNA samples from the tails of three animals in each genotype group were prepared and PCR products from each individual animal (not pooled) were subsequently sequenced. The results are presented in Table 2, which demonstrates that the genotype of each mouse identified by the tetra-primer ARMS-PCR method correctly matched its genetic sequence information.

**Figure 5:**
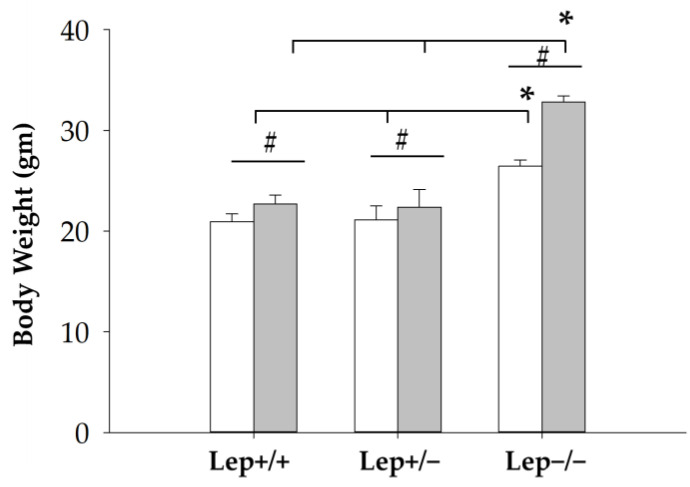
The body weights of mice were measured at ages of 5 (white bar) and 6 (gray bar) weeks. Data represented mean ± SEM of each group: Lep+/+ (n = 7), Lep+/− (n = 5), and Lep−/− (n = 4). Data were analyzed with two-way ANOVA followed by all pairwise multiple comparison Holm-Sidak post hoc test. Statistical significance set at *p* < 0.05; # indicates the statistical significance of body weight within each genotype between weeks of 5 and 6. * indicates the statistical significance of body weight within each week among three different, respectively.

The genotypes identified by the tetra-primer ARMS-PCR were further confirmed by the body weight of the mice. As shown in Figure 5, at ages of week 5 and week 6, *ob*/*ob* mice (Lep−/−) were significantly heavier than the heterozygous or wild-type mice. (Week 5: Wildtype 20.94 ± 0.76; Heterozygous 21.10 ± 1.39; *ob*/*ob* 26.43 ± 0.62, *p* < 0.05; Week 6: Wildtype 22.67 ± 0.91; Heterozygous 22.38 ± 1.77; *ob/ob* 32.8 ± 0.64, *p* < 0.05).

A few studies have reported on how to identify leptin gene mutation(s). Chung et al. utilized polymerase chain reaction-restriction fragment length polymorphisms (PCR-RFLP), which was PCR followed by restriction enzyme digestion of the PCR products to detect leptin mutation [26]. PCR-RFLP is known to be time-consuming and expensive. In addition, PCR-RFLP is only possible when the polymorphism creates or abolishes a restriction enzyme site [27]. A modified method using two separate PCRs with a three-primer mix could differentiate mice with wildtype, heterozygous, and homozygous *ob*/*ob* alleles [27]. However, it was not successful in using one single PCR run to generate fragments because of the ineffective binding of primers at temperatures optimal for mutant and wild-type-specific primers [27]. Oler et al. developed a gel-free SNP detection for leptin mutation [28]. In this method, the annealing of the oligonucleotide complementary to the SNP allele led to the cleavage of the oligonucleotide by a cleavase enzyme. The released 5′ arm fragment then served as an invader, leading to a secondary cleavage reaction to release a fluorescent signal from fluorescence resonance energy transfer (FRET) cassettes [29]. This method uses a commercial Invader assay kit, and the synthesis of FRET cassettes could be expensive under resource-limited settings. In addition, the results were not directly visualizable. Ayabe and colleagues reported a tetra-primer ARMS-PCR to detect leptin mutation [30]. However, a 4% agarose gel was used due to the small size differences between the amplicons of the C allele and the T allele (243 bp for the C allele and 208 bp for the T allele, with only a 35 bp difference in size between the two). Both amplicons ran between 200 bp and 300 bp DNA ladder markers on the gel, making it challenging to distinguish the relative positions of the two bands, i.e., wild-type mice from *ob*/*ob* mice (both genotypes have two bands on the gel).

In this report, we developed a tetra-primer ARMS-PCR to identify three different genotypes using as little as 12.5 µL of PCR reaction. The genotypes corresponding to different patterns of DNA amplicons have been verified by sequence analysis, the gold standard method. In our method, the size difference of amplicons between C and T alleles is 82 bp. Regarding the position of each amplicon, the T allele amplicon is above a 100 bp DNA ladder, the C allele amplicon is above a 200 bp DNA ladder, and the outer band is above a 300 bp DNA ladder, respectively. The three genotype patterns were clearly visible, well separated, and distinguishable under a 1.5% agarose gel using a Quick-Load^®^ 100 bp DNA Ladder as the reference. The process of PCR reaction together with gel electrophoresis took less than 2 h for a total of 24 samples. Therefore, our method can potentially be adapted for high-throughput analysis purposes.

## 4. Conclusions

We have developed a tetra-primer ARMS-PCR to detect a single-point mutation in leptin gene. The method is quick, easy to conduct, and suitable for genotyping *ob*/*ob* mice and their wild-type controls for obesity and diabetes-related research. Also, it is cost-effective to maintain the appropriate size of animal colonies in settings with constrained resources.

## Figures and Tables

**Table 1:** Tetra-primer ARMS-PCR primers for the detection of obese gene point mutation.

Name	Sequences	Length of Expected PCR Amplicons
Forward Outer (FO)	5′-GGTCACTGGCTTGGACTTCA-3′	Between FO and RO pair: 369 bp
Reverse Outer (RO)	5′-TGATTCTTGGGAGCCTGGTGGCCTTTGA-3′	Between FI and RO pair: 253 bp (C allele, wildtype)
Forward Inner (FI)	5′-TGCAGATAGCCAATGACCTGGAGAATCGCC-3′	Between FO and RI pair: 171 bp (T allele, mutant)
Reverse Inner (RI)	5′-AAGGCCAGCAGATGGAGGAGGTCGCA-3′	

Footnote: Forward Outer and Reverse Outer are positioned in the wildtype sequence on the DNA region outside the single point leptin mutation. The bases in red in Forward and Reverse Inner primers indicate a purposely introduced additional mismatch at position −2 from the 3′ terminal of the same allele-specific primer to increase the specificity of the PCR reaction [21].

**Table 2:** Sequencing results to verify the genotypes of mice identified by the tetra-primer ARMS-PCR.

Animal ID	Sequencing Results	Allele	Genotype
129	CTGGAGAATCTCTGCGACCTC	T allele	Lep+/−, heterozygous
129	CTGGAGAATCGCCGAGACCTC	C allele	
136	CTGGAGAATCTCTGCGACCTC	T allele	Lep+/−, heterozygous
136	CTGGAGAATCGCCGAGACCTC	C allele	
137	CTGGAGAATCTCTGCGACCTC	T allele	Lep+/−, heterozygous
137	CTGGAGAATCGCCGAGACCTC	C allele	
128	CTGGAGAATCGCCGAGACCTC	C allele	Lep+/+, wildtype
130	CTGGAGAATCGCCGAGACCTC	C allele	Lep+/+, wildtype
131	CTGGAGAATCGCCGAGACCTC	C allele	Lep+/+, wildtype
138	CTGGAGAATCTCTGCGACCTC	T allele	Lep−/−, ob/ob
139	CTGGAGAATCTCTGCGACCTC	T allele	Lep−/−, ob/ob
135	CTGGAGAATCTCTGCGACCTC	T allele	Lep−/−, ob/ob

Footnote: Sequencing results of the 253 bp and 171 bp amplicons were from nine mice. Gel bands corresponding to 253 bp or 171 bp from Lep+/+, Lep+/− or Lep−/− mice, were cut out, purified from the gel with Zymoclean Gel DNA Recovery Kit (Zymo Research, Irvine, CA, USA), and sequenced using an Applied Biosystem 3730 DNA Analyzer (Waltham, MA, USA) following Applied Biosystems BigDye Terminator v3.1 recommendations. Sequencing results of amplicon band size of 253 bp were produced using Reverse Outer as the primer. Sequencing results of amplicon band size of 171 bp were produced using Forward Outer as the primer. Red colored letter: T (Thymine); C (Cytosine).

## Data Availability

The data presented in this study are available in the article. Body weight data presented in this study are available on request from the corresponding authors.
